# Ancient Metabolisms of a Thermophilic Subseafloor Bacterium

**DOI:** 10.3389/fmicb.2021.764631

**Published:** 2021-12-01

**Authors:** Amy R. Smith, Ryan Mueller, Martin R. Fisk, Frederick S. Colwell

**Affiliations:** ^1^Department of Science, Mathematics, and Computing, Bard College at Simon’s Rock, Great Barrington, MA, United States; ^2^Department of Marine Chemistry and Geochemistry, Woods Hole Oceanographic Institution, Woods Hole, MA, United States; ^3^College of Earth, Ocean, and Atmospheric Sciences, Oregon State University, Corvallis, OR, United States

**Keywords:** metabolism, carbon fixation, acetogenesis, bacteria, seafloor, hydrogen, amino acid, clostridia

## Abstract

The ancient origins of metabolism may be rooted deep in oceanic crust, and these early metabolisms may have persisted in the habitable thermal anoxic aquifer where conditions remain similar to those when they first appeared. The Wood–Ljungdahl pathway for acetogenesis is a key early biosynthetic pathway with the potential to influence ocean chemistry and productivity, but its contemporary role in oceanic crust is not well established. Here, we describe the genome of a novel acetogen from a thermal suboceanic aquifer olivine biofilm in the basaltic crust of the Juan de Fuca Ridge (JdFR) whose genome suggests it may utilize an ancient chemosynthetic lifestyle. This organism encodes the genes for the complete canonical Wood–Ljungdahl pathway, but is potentially unable to use sulfate and certain organic carbon sources such as lipids and carbohydrates to supplement its energy requirements, unlike other known acetogens. Instead, this organism may use peptides and amino acids for energy or as organic carbon sources. Additionally, genes involved in surface adhesion, the import of metallic cations found in Fe-bearing minerals, and use of molecular hydrogen, a product of serpentinization reactions between water and olivine, are prevalent within the genome. These adaptations are likely a reflection of local environmental micro-niches, where cells are adapted to life in biofilms using ancient chemosynthetic metabolisms dependent on H_2_ and iron minerals. Since this organism is phylogenetically distinct from a related acetogenic group of Clostridiales, we propose it as a new species, *Candidatus* Acetocimmeria pyornia.

## Introduction

The extrusive layer of igneous oceanic crust is one of Earth’s largest microbial habitats, and, though estimates vary widely, this habitat could contain up to 75% of the total carbon biomass on Earth (~200Pg of carbon; Pg=10^15^g; Whitman et al., 1998; Heberling et al., 2010; Kallmeyer et al., 2012). Water–rock reactions common to the deep crust support active subsurface chemosynthetic microbial communities (Edwards et al., 2011; Lever et al., 2013) through the production of reduced compounds such as molecular hydrogen and labile organic carbon. These products are subsequently vented to the seafloor and may significantly increase secondary productivity and carbon cycling in the deep ocean (Mason et al., 2009; McCarthy et al., 2010). Due to the logistical difficulties of deep biosphere sampling and the lack of cultivation strategies for autochthonous microbes, little is known about the rates of chemosynthesis and the corresponding metabolic capabilities of organisms from this habitat. The Juan de Fuca Ridge (JdFR) flank and its permanent subsurface microbial observatories represent one of the few places in the world where access to these types of environments is available.

Recently, it was reported that the Wood–Ljungdahl pathway for acetogenesis may be a significant chemosynthetic pathway within the oceanic crustal aquifer (Jungbluth et al., 2017; Smith et al., 2019). In deep oligotrophic environments like the JdFR aquifer (Lin et al., 2012), annual photosynthetically derived carbon inputs are low; thus, chemosynthetic metabolisms such as acetogenesis are hypothesized to play an important role. Acetogenesis may have first appeared in the Earth’s crust as one of the first carbon fixation pathways and remained the dominant form of primary production until the evolution of photosynthesis (Ragsdale and Pierce, 2008; Nitschke and Russell, 2013; Martin, 2020). Thus, acetogenesis may remain a key metabolic strategy in deep crustal aquifers, where contemporary conditions can often mirror those of the primordial state on early Earth (hot reducing fluids reacting with igneous minerals in oceanic crust).

The Wood–Ljungdahl pathway is a metabolically versatile pathway that can be used for biosynthesis by producing acetyl-CoA, or for energy conservation when completing the pathway and excreting acetate. Acetogens may use either mode of this metabolic pathway, switching between them depending on the needs of the organism (Pierce et al., 2008; Lever, 2012). Additional energy could be gained from this pathway by utilizing sodium or proton pumps to create a proton motive force that drives subsequent ATP generation using ATP synthase (Schuchmann and Müller, 2014). This pathway has two branches, the carbonyl and the methyl branch, which both rely on molecular hydrogen (H_2_) to reduce and condense two molecules of CO_2_ to form acetyl-CoA. Formate, CO, and methanol are also substrates that can be used. As a chemosynthetic pathway, the acetyl-CoA end-product is a central metabolic intermediate that can be used in multiple anabolic pathways, such as lipid, carbohydrate, and amino acid biosynthesis (Pierce et al., 2008; Ragsdale, 2008).

Conditions in deep crustal aquifers are particularly favorable for organisms relying on the Wood–Ljungdahl pathway for carbon fixation. Molecular hydrogen is the key reductant of this pathway, and its high abundance in deep crustal aquifers is a result of chemical reactions between water and iron-bearing minerals. Bicarbonate (HCO_3_^−^), the other key reactant of this pathway, is also known to be abundant in the oceanic crust (Takai et al., 2004; Brazelton et al., 2006; Ver Eecke et al., 2012; Lin et al., 2014). Although acetogenesis is less thermodynamically favorable than methanogenesis, which also uses H_2_ and CO_2_ (or HCO_3_^−^), acetogens have the advantage of requiring a smaller set of enzymes for biosynthesis and energy generation *via* the Wood–Ljungdahl pathway. This lowers the overall energetic cost of metabolism and may allow acetogens to compete more effectively for resources with methanogens (Ragsdale and Pierce, 2008; Lever, 2012; Nitschke and Russell, 2013).

Since the production of acetate yields little net energy, acetogens often utilize additional metabolisms such as sulfate reduction and heterotrophy to supplement their energy needs (Drake et al., 1997; Ragsdale and Pierce, 2008; Lever, 2012; Thór Marteinsson et al., 2012; Schuchmann and Müller, 2014). *Moorella thermoacetica* (Fontaine et al., 1942; Pierce et al., 2008), *Candidatus* Desulforudis audaxviator (Chivian et al., 2008), and *Desulfopertinax hafniense* (Nonaka et al., 2006) are three terrestrial acetogens who have had their complete genome sequenced, and each encodes additional energy-generating pathways uniquely suited to their needs. *Moorella thermoacetica* is a common acetogen that uses carbohydrates and a wide variety of other substrates for energy (Fontaine et al., 1942; Pierce et al., 2008). *Candidatus* Desulforudis audaxviator is a deep subsurface microorganism that can use H_2_ derived from radiolytic processes and is able to reduce sulfate and fix nitrogen (Chivian et al., 2008). *Desulfitobacterium hafniense* is metabolically versatile and is able to use dehalogenation and a multitude of organic and inorganic electron acceptors and donors to generate energy (Nonaka et al., 2006). These divergent strategies are likely dependent largely on the unique environmental conditions in which they thrive. In the marine subsurface, inorganic electron donors and acceptors are expected to comprise the primary redox couples, much like the deep terrestrial subsurface where *Ca.* D. audaxviator is known to live. However, peptides and amino acids have also been reported both in aquifer fluid and adsorbed to mineral surfaces, leaving open the possibility of organoheterotrophic processes in this environment (Lin et al., 2015).

The following report compares the genome characteristics of a MAG obtained from an olivine biofilm in the deep subsurface of oceanic crust to the genomes of these three known acetogens from terrestrial environments. The purpose of this study was to determine the similarities and differences between deep marine and terrestrial acetogens to inform the adaptations required for life in a deep crustal aquifer. Based on the inferred ecology and metabolism of this organism, we propose this novel subseafloor acetogen as *Candidatus* Acetocimmeria pyornia (aceto=acetogen; Cimmeria=dweller of dark land beyond the ocean; pyornia, short for Pyornkrachzark, or “Rock Biter,” character from the 1984 film *The NeverEnding Story*).

## Materials and Methods

### *In situ* Colonization of Olivine in the JdFR Aquifer

Olivine and other igneous minerals and glasses were incubated in 3.5-mya oceanic crust for 4years to investigate the properties of surface-colonizing microbial communities from the JdFR aquifer. These ~2-mm-sized mineral grains were emplaced in flow cells at 275–287m below seafloor (mbsf) in International Ocean Drilling Program Hole 1301A (47° 45.210' N, 127° 45.833' W; 2,667m below sea level) on the eastern flank of the JdFR (Fisher et al., 2005; Smith et al., 2011, 2016). Details of incubation and sample retrieval have been reported previously and are only briefly summarized here (Smith et al., 2011, 2016). After the 4-year incubation in Hole 1301A, recovered minerals were frozen at −40°C.

### Genomic DNA Extraction and Metagenome Sequencing

Extraction and amplification methods were published previously (Smith et al., 2011, 2016). Briefly, genomic DNA was extracted from ~500mg olivine using a modified protocol for the FastDNA Spin Kit for Soil (MP Biomedicals Catalog #116560200) as described previously (Wang and Edwards, 2009; Smith et al., 2016). Genomic DNA extracted from the two most common olivine mineral phases (forsterite and Fo_90_ olivine) was pooled to produce the olivine metagenome (a total of 50–70ng of DNA). The olivine genomic DNA extract was sent to the Census of Deep Life sequencing facility at Marine Biological Laboratory in Woods Hole, MA, United States. Metagenomic sequencing was carried out on an Illumina Hi-Seq1000 instrument with 2×101bp paired-end sequencing and dedicated-read indexing. Sequence reads were de-multiplexed using the Consensus Assessment of Sequence and Variance (CASAVA) 1.8.2 (Illumina). A total of 84 million reads with average length of 108 bases were produced for the olivine metagenome, totaling 9.8 gigabases of sequence data.

### Sequence Quality Check, Assembly, and Binning

Sequence quality check, assembly, and binning protocols were reported previously (Smith et al., 2019) and are summarized briefly again here. Raw sequence files were quality-filtered using String Graph Assembler (SGA)‘s preprocessing pipeline (Simpson and Durbin, 2012; Smith et al., 2019). High-quality mate pair sequence reads were assembled using the Iterative deBruin Graph Assembler—Uneven Depth (IDBA-UD) program (Peng et al., 2012) with the following specifications: minimum contig length of 450 bases and iterative kmer values from 45 to 69, stepwise by 4. The assembly with kmer length of 65 was chosen for use in analytical subsequent steps based on optimal values of n50 and maximum contig size.

VizBin (Laczny et al., 2014), a Java-based genomic binning program, which uses nonlinear dimension reduction of genomic signatures (i.e., nucleotide frequency) to assign contigs to taxonomic bins, was used to separate contig groups from the full assembly into reduced genome bins based on sequence similarity (see Smith et al., 2019). A total of 12 bins were detected in the olivine metagenome assembly, each of which was exported for manual bin reconstruction. The *Ca. A. pyornia* metagenome-assembled genome (MAG JdFRolivine-5) is described here. All genome MAGs from the olivine community were uploaded to the Metagenomics Analysis Server (MG-RAST) for initial analyses and comparison of genome features. CheckM (Parks et al., 2015) was used to verify taxonomic purity (3.96% contamination) and completeness (91.58%) of MAGs using a total of 43 universal marker genes present in complete bacterial genomes. The genome is publicly available on NCBI’s website with accession #SESX00000000, biosample SAMN10863484.

### Phylogenetic Tree Construction

A number of 16S rRNA genes were included within the JdFRolivine-5 MAG (subsequently defined as the *Ca.* A. pyornia MAG); therefore, we used several methods to determine phylogenetic relatedness of this genome’s sequences to other references. First, we employed whole genome phylogenetic tree construction in The Department of Energy Systems Biology Knowledgebase (KBase; Arkin et al., 2018) with Species Tree v.1.1.2. We used a set of 49 concatenated core universal marker genes to construct the genome tree. Input genomes are combined with closely related genomes from the RefSeq database from KBase, and a curated multiple sequence alignment of a subset of 49 COG domains is used to determine relatedness. The alignments are trimmed, concatenated, and then used to a construct phylogenetic tree with maximum likelihood phylogeny. We set a threshold of 20 nearby genomes to construct the tree and did not remove any genomes. We performed FastANI v.0.1.3 (Goris et al., 2007; Jain et al., 2018), or whole-genome Average Nucleotide Identity (ANI), in KBase to determine relatedness to these nearby genomes.

We also compiled phylogenetic results from PhyloPythia, CheckM, MG-RAST, Kyoto Encyclopedia of Genes and Genomes (KEGG), and the PyroTag sequencing of olivine community DNA (Smith et al., 2016, 2019) to identify the relatedness of known species. In order to include more environmental sequences in our phylogenetic analysis, we chose the *ffh* gene sequence, another highly conserved, single copy phylogenetic marker gene (Sunagawa et al., 2013) that can be used in lieu of the 16S rRNA gene, to construct a maximum likelihood tree based on the Tamura-Nei model in MEGA5 (Tamura et al., 2011; Supplementary Figure 1). This tree also includes related sequences obtained from the same sample: JdFRolivine-7, JdFRolivine-9, and the organism reported here, *Ca.* Acetocimmeria pyornia (identified as MAG JdFRolivine-5; Smith et al., 2019). The initial *ffh* trees were obtained through the Neighbor-Join (Saitou and Nei, 1987) and BioNJ (Gascuel, 1997) algorithms. These algorithms were applied to a matrix of pairwise distances that were estimated by maximum composite likelihood. Positions that represented gaps or missing data were eliminated from the analysis. A total of 264 positions were used to construct the phylogenetic tree.

### Genome Annotation and Metabolic Pathway Reconstruction

Gene sequences of MAGs obtained using Prodigal v2.6.0 were uploaded to the KEGG and annotated using BlastKOALA (Kanehisa et al., 2016). The reconstructed metabolism for the *Ca.* A. pyornia MAG was used to assess the potential for carbon fixation (Table 1), determine likely energy sources, and explore mineral or surface-based metabolic properties that may yield clues as to how this organism survives in JdFR aquifer biofilms. The resulting metabolic pathway reconstruction was compared against KEGG genome annotations of closely related acetogens *Ca.* Desulforudis audaxviator (Chivian et al., 2008), *Moorella thermoacetica* (Pierce et al., 2008), and *Desulfitobacterium hafniense* (Nonaka et al., 2006; Tables 2–4; Supplementary Tables 1–3).

**Table 1:** Kyoto Encyclopedia of Genes and Genomes (KEGG) annotations of contig-65_203 containing the *acs* gene cluster of the Wood–Ljungdahl pathway for carbon fixation.

Gene ID for Contig-65_203	Gene definition	KO best match	Second best match
contig-65_203_1	*Unassigned*	*None*	
contig-65_203_2	*Unassigned*	*None*	
contig-65_203_3	cooS; carbon-monoxide dehydrogenase catalytic subunit [EC:1.2.7.4]	K00198	
contig-65_203_4	acsB; acetyl-CoA synthase [EC:2.3.1.169]	K14138	
contig-65_203_5	cdhE; acetyl-CoA decarbonylase/synthase complex subunit gamma [EC:2.1.1.245]	K00197	
contig-65_203_6	*Unassigned*	*None*	
contig-65_203_7	cooC; CO dehydrogenase maturation factor	K07321	
contig-65_203_8	cdhD; acetyl-CoA decarbonylase/synthase complex subunit delta [EC:2.1.1.245]	K00194	
contig-65_203_9	acsE; 5-methyltetrahydrofolate corrinoid/iron sulfur protein methyltransferase [EC:2.1.1.258]	K15023	
contig-65_203_10	hdrA; heterodisulfide reductase subunit A [EC:1.8.98.1]	K03388	
contig-65_203_11	*Unassigned*	*None*	K14127
contig-65_203_12	*Unassigned*	*None*	
contig-65_203_13	metF; methylenetetrahydrofolate reductase (NADPH) [EC:1.5.1.20]	K00297	
contig-65_203_14	*Unassigned*	*None*	K17836
contig-65_203_15	E4.2.1.2AA; fumarate hydratase subunit alpha [EC:4.2.1.2]	K01677	
contig-65_203_16	E4.2.1.2AB; fumarate hydratase subunit beta [EC:4.2.1.2]	K01678	
contig-65_203_17	ME2; malate dehydrogenase (oxaloacetate-decarboxylating) [EC:1.1.1.38]	K00027	
contig-65_203_18	sdhC; succinate dehydrogenase/fumarate reductase, cytochrome b subunit	K00241	
contig-65_203_19	sdhD; succinate dehydrogenase/fumarate reductase, membrane anchor subunit	K00242	K00241
contig-65_203_20	sdhA; succinate dehydrogenase/fumarate reductase, flavoprotein subunit [EC:1.3.5.1 1.3.5.4]	K00239	
contig-65_203_21	sdhB; succinate dehydrogenase/fumarate reductase, iron-sulfur subunit [EC:1.3.5.1 1.3.5.4]	K00240	
contig-65_203_22	sucC; succinyl-CoA synthetase beta subunit [EC:6.2.1.5]	K01903	
contig-65_203_23	sucD; succinyl-CoA synthetase alpha subunit [EC:6.2.1.5]	K01902	
contig-65_203_24	*Unassigned*	*None*	K15876
contig-65_203_25	*Unassigned*	*None*	K01181

Contig-65_203 contains 25 genes, here numbered contig-65_203_1 – contig-65_203_25. Some genes do not have matching orthologous proteins in the KEGG database, and the gene or function is therefore unassigned. Some of these matches do have “second best” matches, which may be used to infer the closest function. KO, KEGG Orthology.

**Table 2:** Complete pathways of carbon fixation and energy metabolism in *Candidatus* A. pyornia (*Ca.* Apy) and three closely related acetogens as determined by the KEGG pathway module.

KEGG Gene	Discrete pathway	Organism			
Energy metabolism		Mta	Dau	Dsy	*Ca.* Apy
Carbon fixation					
M00377	Reductive acetyl-CoA pathway (Wood–Ljungdahl pathway)	(+)	**+**	**+**	**+**
M00579	Phosphate acetyltransferase-acetate kinase pathway, acetyl-CoA≥acetate	**+**		**+**	**+**
Nitrogen metabolism					
M00175	Nitrogen fixation, nitrogen≥ammonia		**+**	**+**	
M00530	Dissimilatory nitrate reduction, nitrate≥ammonia			**+**	
Methane metabolism					
M00422	Acetyl-CoA pathway, CO_2_≥acetyl-CoA		**+**		
Sulfur metabolism					
M00596	Dissimilatory sulfate reduction, sulfate≥H_2_S		**+**		(−)

Closely related known acetogens: Mta, *Moorella thermoacetica*, Dau, *Ca. Desulforudis audaxviator*, and Dsy, *Desulfitobacterium hafniense*. +, complete pathway, (+), known complete pathways not identified through the KEGG module. (−), near-complete, with at least one gene.

**Table 3:** Complete pathways of carbohydrate and lipid metabolism in *Ca.* A. pyornia (*Ca.* Apy) and two closely related acetogens as determined by the KEGG pathway module.

KEGG Gene	Discrete pathway	Organism			
Energy metabolism		Mta	Dau	Dsy	*Ca.* Apy
Carbohydrate and lipid metabolism					
Central carbohydrate metabolism					
M00001	Glycolysis (Embden–Meyerhof pathway), glucose≥pyruvate	**+**	**+**	**+**	(−)
M00002	Glycolysis, core module involving three-carbon compounds	**+**	**+**	**+**	**+**
M00003	Gluconeogenesis, oxaloacetate≥fructose-6P	**+**	**+**	**+**	(−)
M00010	Citrate cycle, first carbon oxidation, oxaloacetate≥2-oxoglutarate	**+**			(−)
M00307	Pyruvate oxidation, pyruvate≥acetyl-CoA			**+**	
M00007	Pentose phosphate pathway, non-oxidative phase, fructose 6P≥ribose 5P	**+**	**+**	**+**	**+**
M00308	Semi-phosphorylative Entner–Doudoroff pathway, gluconate≥glycerate-3P				(−)
M00005	PRPP biosynthesis, ribose 5P≥PRPP	**+**	**+**	**+**	**+**
Other carbohydrate metabolism					
M00741	Propanoyl-CoA metabolism, propanoyl-CoA≥succinyl-CoA				(−)
M00549	Nucleotide sugar biosynthesis, glucose≥UDP-glucose			**+**	(−)
Fatty acid metabolism					
M00082	Fatty acid biosynthesis, initiation				(−)
M00083	Fatty acid biosynthesis, elongation	**+**	**+**	**+**	**+**
M00086	beta-Oxidation, acyl-CoA synthesis			**+**	
Lipid metabolism					
M00093	Phosphatidylethanolamine (PE) biosynthesis, PA≥PS≥PE			**+**	
Terpenoid backbone biosynthesis					
M00096	C5 isoprenoid biosynthesis, non-mevalonate pathway	**+**			**+**
M00364	C10-C20 isoprenoid biosynthesis, bacteria				**+**

Closely related known acetogens: Mta, *Moorella thermoacetica*, Dau, *Ca. Desulforudis audaxviator*, and Dsy, *Desulfitobacterium hafniense*. +, complete pathway, (+), known complete pathways not identified through the KEGG module. (−), near-complete, with at least one gene.

**Table 4:** Complete pathways of nucleotide and amino acid metabolism in *Ca.* A. pyornia (*Ca.* Apy) and two closely related acetogens as determined by the KEGG pathway module.

KEGG Gene	Discrete pathway	Organism			
Energy metabolism		Mta	Dau	Dsy	*Ca.* Apy
Nucleotide and amino acid metabolism					
Purine metabolism					
M00048	Inosine monophosphate biosynthesis, PRPP + glutamine≥IMP		**+**		(−)
M00049	Adenine ribonucleotide biosynthesis, IMP≥ADP,ATP	**+**	**+**	**+**	**+**
M00050	Guanine ribonucleotide biosynthesis IMP≥GDP,GTP	**+**	**+**	**+**	**+**
Pyrimidine metabolism					
M00051	Uridine monophosphate biosynthesis, glutamine (+PRPP)≥UMP				(−)
M00052	Pyrimidine ribonucleotide biosynthesis, UMP≥UDP/UTP,CDP/CTP	**+**	**+**	**+**	**+**
Serine and threonine metabolism					
M00018	Threonine biosynthesis, aspartate≥homoserine≥threonine	**+**	**+**	**+**	**+**
Cysteine and methionine metabolism					
M00021	Cysteine biosynthesis, serine≥cysteine	**+**	**+**	**+**	**+**
Branched-chain amino acid metabolism					
M00019	Valine/isoleucine biosynthesis, pyruvate≥valine/2-oxobutanoate≥isoleucine	**+**	**+**	**+**	**+**
M00570	Isoleucine biosynthesis, threonine≥2-oxobutanoate≥isoleucine		**+**	**+**	**+**
M00432	Leucine biosynthesis, 2-oxoisovalerate≥2-oxoisocaproate	**+**	**+**	**+**	**+**
Lysine metabolism					
M00526	Lysine biosynthesis, DAP dehydrogenase pathway, aspartate≥lysine			**+**	(−)
M00527	Lysine biosynthesis, DAP aminotransferase pathway, aspartate≥lysine	**+**	**+**	**+**	**+**
Arginine and proline metabolism					
M00015	Proline biosynthesis, glutamate≥proline	**+**	**+**	**+**	**+**
M00028	Ornithine biosynthesis, glutamate≥ornithine	**+**	**+**	**+**	(−)
Histidine metabolism					
M00045	Histidine degradation, histidine≥N-formiminoglutamate≥glutamate				(−)
M00026	Histidine biosynthesis, PRPP≥histidine	**+**	**+**	**+**	**+**
Aromatic amino acid metabolism					
M00022	Shikimate pathway, phosphoenolpyruvate+erythrose-4P≥chorismate	**+**	**+**	**+**	**+**
M00023	Tryptophan biosynthesis, chorismate≥tryptophan	**+**	**+**		**+**
M00024	Phenylalanine biosynthesis, chorismate≥phenylalanine				(−)
M00025	Tyrosine biosynthesis, chorismate≥tyrosine				(−)
Cofactor and vitamin biosynthesis					
M00127	Thiamine biosynthesis, AIR≥thiamine-P/thiamine-2P	**+**			**+**
M00125	Riboflavin biosynthesis, GTP≥riboflavin/FMN/FAD				**+**
M00115	NAD biosynthesis, aspartate≥NAD		**+**	**+**	
M00119	Pantothenate biosynthesis, valine/L-aspartate≥pantothenate			**+**	(−)
M00120	Coenzyme A biosynthesis, pantothenate≥CoA	**+**	**+**	**+**	**+**
M00123	Biotin biosynthesis, pimeloyl-ACP/CoA≥biotin		**+**		
M00122	Cobalamin biosynthesis, cobinamide≥cobalamin				(−)
M00140	C1-unit interconversion, prokaryotes	**+**		**+**	**+**
M00141	C1-unit interconversion, eukaryotes		**+**		
Polyamine biosynthesis					
M00133	Polyamine biosynthesis, arginine≥agmatine≥putrescine≥spermidine	**+**	**+**		**+**

Closely related known acetogens: Mta, *Moorella thermoacetica*, Dau, *Ca. Desulforudis audaxviator*, and Dsy, *Desulfitobacterium hafniense*. +, complete pathway, (+), known complete pathways not identified through the KEGG module. (−), near-complete, with at least one gene.

Genes not covered by pathway analysis were searched using the KEGG-annotated genome data obtained through BlastKOALA for all contigs. Genes necessary for completion of near-complete pathways or those that may be involved in alternative pathways were searched and identified. Information regarding other genes relating to biofilm formation, chemotaxis and flagella, pilus formation, metal transport or utilization, and others was gathered from the annotated genome.

## Results

### Evolutionary Relatedness

**Figure 1:**
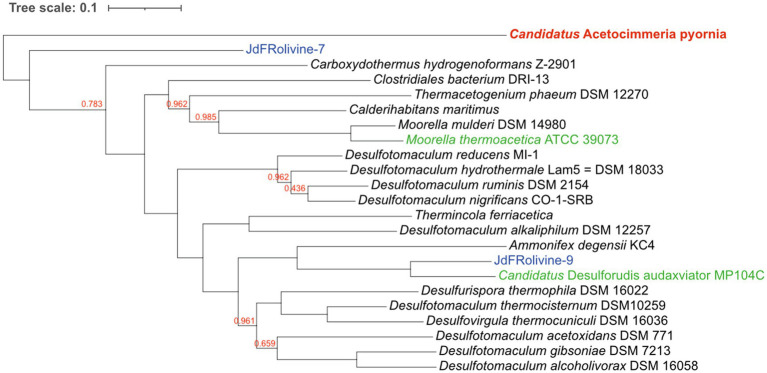
Whole genome phylogenetic tree for *Ca.* Acetocimmeria pyornia and closest genomic relatives. Branches with no bootstrap numbers are 1.000. Branches whose bootstrap numbers are less than 1.000 are indicated on the branch.

The evolutionary relationships of *Ca.* A. pyornia indicate this organism represents a novel acetogen within the bacterial Class Clostridia (Figure 1; Supplementary Figure 1). *Candidatus* A. pyornia is placed within an “acetogen” cluster which includes *M. thermoacetica* (Pierce et al., 2008), *Ca.* D. audaxviator (Chivian et al., 2008), and *D. hafniense* (Nonaka et al., 2006; Supplementary Figure 1). FastANI analysis did not return results as there was <80% whole genome nucleotide similarity between *Ca.* A. pyornia and all nearby available genomes. An ANI percentage of ≥95% is considered the same species as the reference genome (Jain et al., 2018); thus, *Ca.* A. pyornia is at least a new species and most likely a separate genus of Clostridia.

### Genome Attributes

The genome of *Ca.* A. pyornia was predicted to be 91.5% complete, with 3.96% contamination detected (Smith et al., 2019). Of 2,725 predicted coding sequences, 1,446 genes were annotated to a given KEGG orthologous functional group (KO number). *Candidatus* A. pyornia’s genome summary statistics (i.e., genome size and number of protein-encoding genes) were more similar to those of the closely related acetogens *Ca.* D. audaxviator and *M. thermoacetica* than to *D. hafniense*. *Desulfopertinax hafniense*’s genome is roughly twice the size with double the amount of protein-encoding genes (Nonaka et al., 2006). The G+C content, which can reflect environmental factors, was similar among all acetogens in this study (~50%) with the exception of *Ca.* D. audaxviator, whose G+C content is much higher (60.9%; Chivian et al., 2008). When *Ca.* A. pyornia’s genome attributes from BlastKOALA were compared to the seven other high-quality bacterial genomes (>90% complete and <5% contamination; Bowers et al., 2017) from the same community, *Ca.* A. pyornia had the highest percentage of genes for amino acid metabolism and respiration and lowest percentage of carbohydrate metabolism genes. There were 21 protease genes and 15 peptidase genes identified in the *Ca.* A. pyornia genome.

### Wood–Ljungdahl Pathway and the acs Gene Cluster

**Figure 2:**
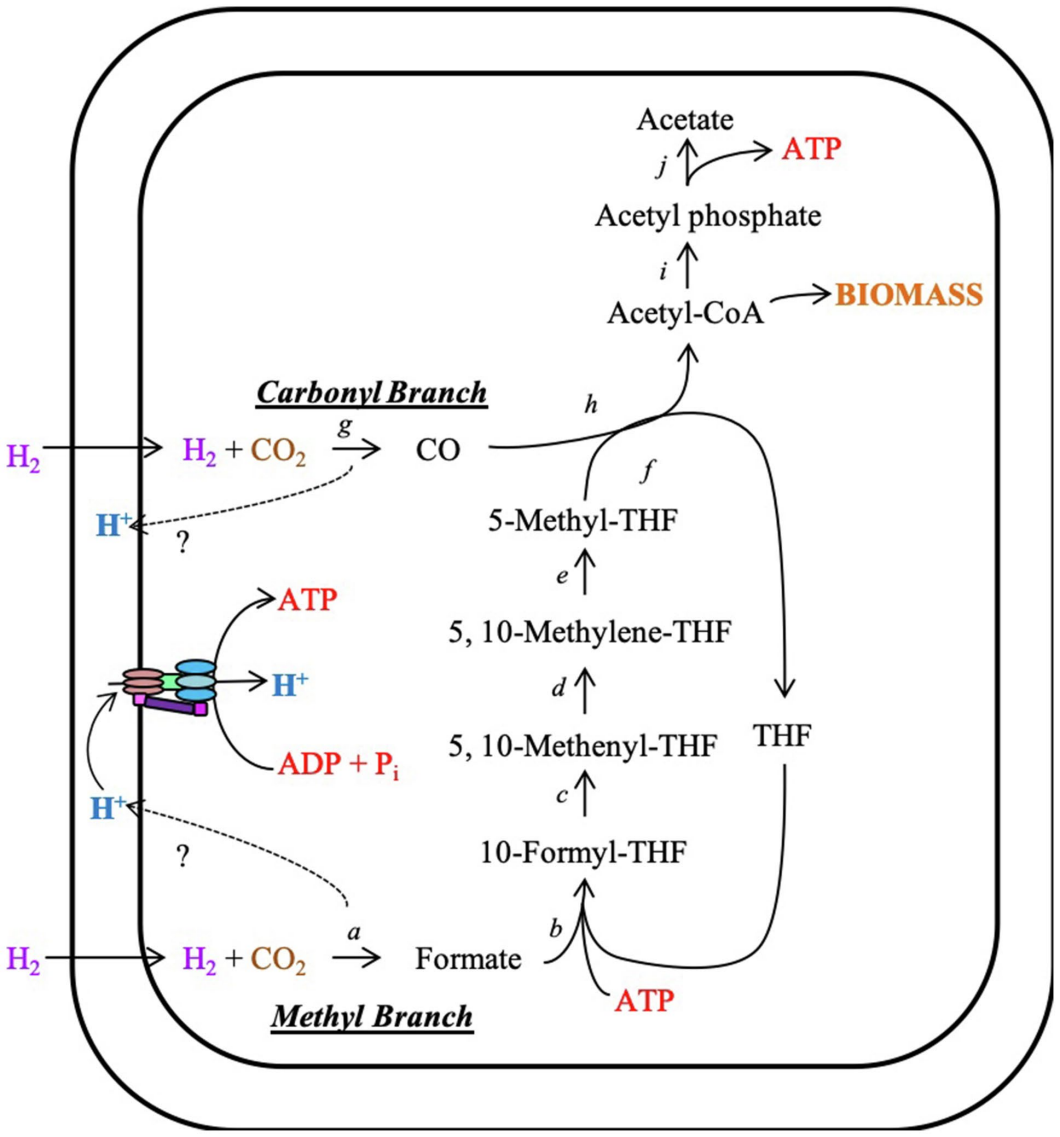
The Wood–Ljungdahl pathway for carbon fixation. The acetogenic Wood–Ljungdahl pathway (or acetyl-CoA) pathway for carbon fixation uses molecular hydrogen and carbon dioxide to produce acetate, generating ATP. If needed, acetyl-CoA can be rerouted for biomass production. The enzymes in this pathway are as follows: *a*, formate dehydrogenase; *b*, formyl-H_4_folate synthase; *c*, formyl-H_4_folate cyclohydrolase; *d*, methylene-H_4_folate dehydrogenase; *e*, methylene-H_4_folate reductase; *f*, methyltransferase; *g*, carbon monoxide dehydrogenase; *h*, acetyl-CoA synthase; *i*, phosphotransacetylase; and *j*, acetate kinase. THF (tetrahydrofolate)=H_4_. The genome of *Ca.* A. pyornia also contains the complete phosphate acetyltransferase-acetate kinase pathway.

**Figure 3:**
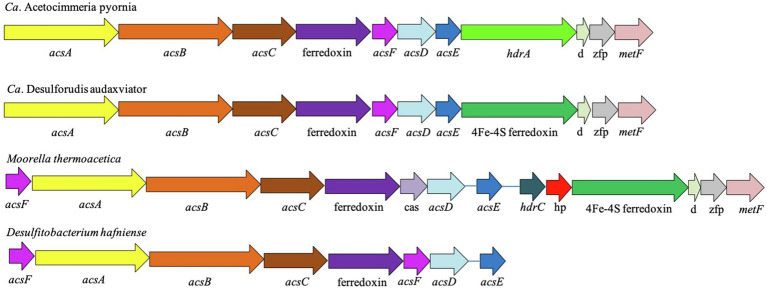
Arrangement of *acs* gene cluster of the Wood–Ljungdahl pathway on contig-65_203 of *Ca.* Acetocimmeria pyornia as compared to closely related acetogens. Descriptions of abbreviated gene IDs and relevant Enzyme Commission (EC) numbers (in brackets) are: *acsA/cooS*, acetyl-CoA synthase alpha subunit/carbon-monoxide dehydrogenase catalytic subunit [EC:1.2.7.4]; *acsB*, acetyl-CoA synthase beta subunit [EC:2.3.1.169]; *acsC/cdhE*, acetyl-CoA decarbonylase/synthase complex subunit gamma [EC:2.1.1.245]; ferredoxin (RefSeq), no KEGG orthology (KO) assigned; *acsF/cooC*, CO dehydrogenase maturation factor; cas, cobyrinic acid a,c-diamide synthase (RefSeq), no KO assigned; *acsD/cdhD*, acetyl-CoA decarbonylase/synthase complex subunit delta [EC:2.1.1.245]; *acsE*, 5-methyltetrahydrofolate corrinoid/iron sulfur protein methyltransferase [EC:2.1.1.258]; *hdrC*, heterodisulfide reductase subunit C; hp, hypothetical protein; *hdrA*, heterodisulfide reductase subunit A [EC:1.8.98.1]; 4Fe-4S ferredoxin (RefSeq), no KO assigned; *d*, methyl-viologen-reducing hydrogenase subunit delta (RefSeq), no KO assigned; zfp, zinc-finger protein (RefSeq), no KO assigned; and *metF*, methylenetetrahydrofolate reductase (NADPH) [EC:1.5.1.20].

*Candidatus* A. pyornia contained the complete Wood–Ljungdahl pathway for acetogenesis (Figure 2). This included genes that code for enzymes and electron transfer agents needed to complete both branches of the pathway. This organism’s genome also contained the complete phosphate acetyltransferase–acetate kinase pathway to transform acetyl-CoA to acetate and recover the energy expended earlier in the pathway (Figure 2). Genes for the carbonyl branch of the Wood–Ljungdahl pathway are contained within an *acs* gene cluster, which is commonly found in acetogens (Figure 3; Table 1). The synteny of the *Ca.* A. pyornia *acs* gene cluster most closely resembled that of *Ca.* D. audaxviator, with only slight differences in the annotations of a 4Fe-4S ferredoxin gene, which is specifically denoted as heterodisulfide reductase A (*hdrA*) in *Ca.* A. pyornia. The *acs* gene clusters of *M. thermoacetica* and *D. hafniense* were less similar to the *acs* gene cluster of *Ca.* A. pyornia due to the addition or loss of genes within the cluster.

### Incomplete TCA Cycle

**Figure 4:**
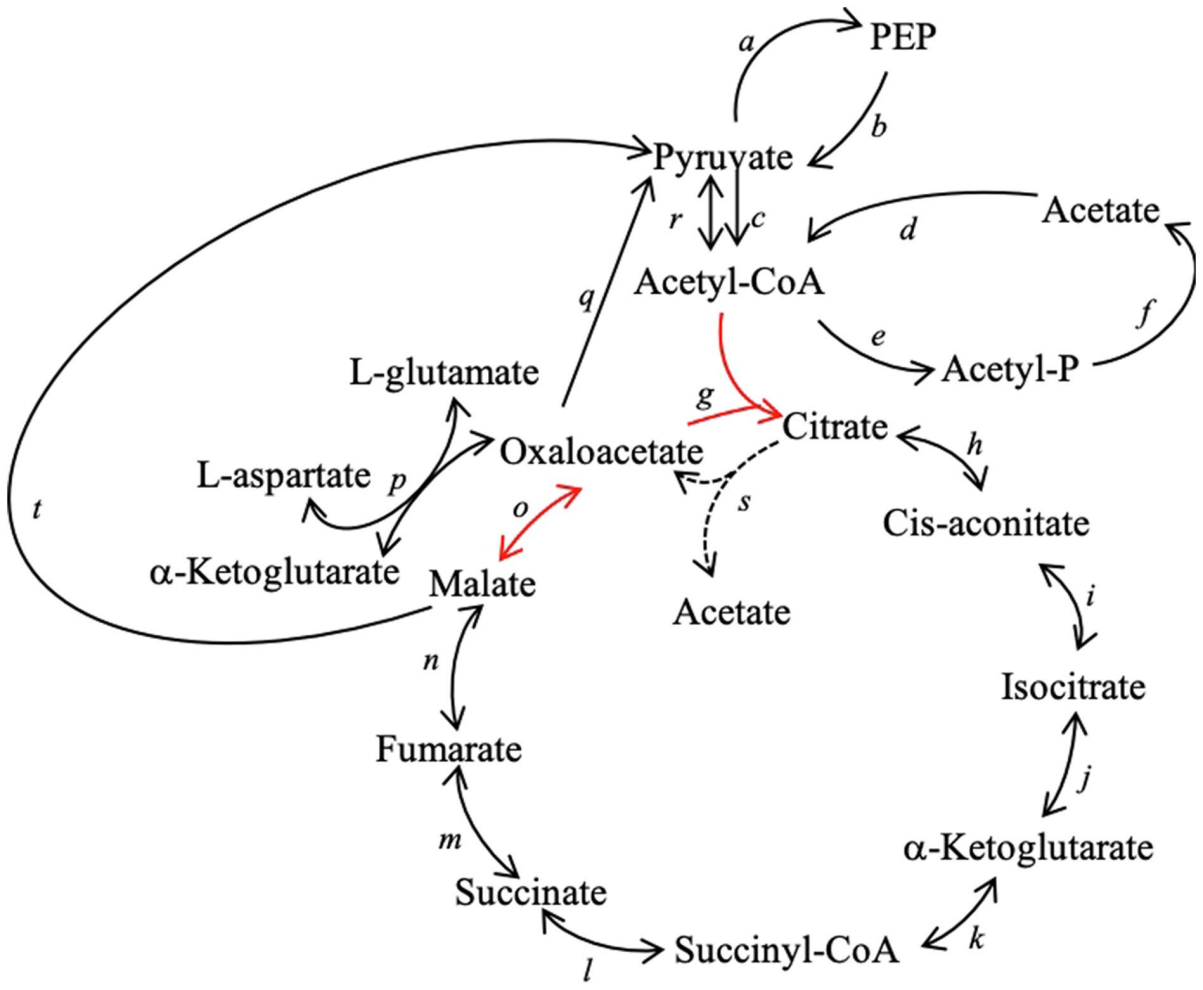
Tricarboxylic Acid (TCA) cycle and related pathways in *Ca.* A. pyornia. *Candidatus* A. pyornia has an incomplete TCA cycle and is missing genes that code for two enzymes: citrate synthase and malate dehydrogenase (red arrows *o* and *g*). Oxaloacetate can still be produced from L-aspartate and 2-oxoglutarate, and oxaloacetate can be decarboxylated to pyruvate. Acetate can be produced through multiple pathways, including the phosphate acetyltransferase-acetate kinase pathway. The enzymes labeled *a* – *s* are as follows: *a*: pyruvate, orthophosphate dikinase, *b*: pyruvate kinase, *c*: pyruvate dehydrogenase, *d*: acetyl-CoA synthase, *e*: putative phosphotransacetylase, *f*: acetate kinase, *g*: citrate synthase, *h* and *i*: aconitase, *j*: isocitrate dehydrogenase, *k*: α-ketoglutarate dehydrogenase, *l*: succinyl-CoA synthetase, *m*: succinate dehydrogenase, *n*: fumarase, *o*: malate dehydrogenase, *p*: aspartate aminotransferase, *q*: oxaloacetate decarboxylase, *r*: pyruvate synthase, *s*: ATP citrate lyase, and *t*: malate dehydrogenase (oxaloacetate-decarboxylating)/malic enzyme. PEP, phosphoenolpyruvate.

*Candidatus* A. pyornia encodes for an incomplete TCA cycle; the genes coding for the enzymes malate dehydrogenase and citrate synthase are missing from this MAG (Figure 4). Malate dehydrogenase transforms malate into oxaloacetate, and citrate synthase uses oxaloacetate and acetyl-CoA to produce citrate and begin the cycle again. However, if portions of the TCA cycle are run in reverse, key biosynthetic intermediates can still be produced from anaplerotic reactions. This genome bin does, however, contain the oxaloacetate-decarboxylating malate dehydrogenase, which is situated in a TCA cluster on the same contig immediately following the *acs* cluster from the Wood–Ljungdahl pathway (Table 1). This portion of the contig also encodes genes for fumarate hydratase, all four succinate dehydrogenase/fumarate reductase subunits, and succinyl-CoA synthetase; however, the oxaloacetate-decarboxylating malate dehydrogenase is likely making pyruvate as a part of anaplerosis, converting it to phosphoenolpyruvate (PEP) and on up the gluconeogenesis/glycolysis pathway to make carbohydrates in order to supplement what it cannot get from the environment. At least one other pathway that results in oxaloacetate formation was identified. The enzyme aspartate aminotransferase can produce oxaloacetate and L-glutamate from L-aspartate and α-ketoglutarate (Figure 4). Oxaloacetate can also be converted to pyruvate using oxaloacetate decarboxylase. The gene for citrate lyase beta subunit was also present; therefore it is possible that other genes not identified could be present in the genome, allowing *Ca.* A. pyornia to produce oxaloacetate from citrate in the reverse direction (Ragsdale and Pierce, 2008). *Candidatus* A. pyornia contains at least two complete pathways for the production of acetate from acetyl-CoA (Figure 4) and can convert pyruvate to PEP and vice versa.

### Oxidative Phosphorylation

*Candidatus* A. pyornia contained all but one gene to construct a complete anaerobic electron transport chain (ETC) for oxidative phosphorylation. We found the near-complete gene set for Complex I, a complete Complex II, and a complete prokaryotic F-type ATPase (Supplementary Table 1). Complex I, or NADH: quinone oxidoreductase (NADH dehydrogenase), is a multi-subunit complex consisting of 14 gene products, 13 of which are present in the *Ca.* A. pyornia genome [subunits A–F and H–N (no G), Supplementary Table 1]. We also found the complete pathway for succinate dehydrogenase, which consists of four genes that code for each of the four subunits of this enzyme, *sdhABCD* (Supplementary Table 1). *Moorella thermoacetica*, *Ca.* D. audaxviator, and *D. hafniense* are all missing the complete gene set to make succinate dehydrogenase (Supplementary Table 1), but all have at least one subunit in their genome. All four acetogens discussed in this manuscript have the F-type ATPase.

### Incomplete Sulfate Reduction Pathway

The *Ca.* A. pyornia genome does not contain genes that allow it to perform dissimilatory sulfate reduction (Table 2). Dissimilatory sulfate reductase genes (*dsrA* and *dsrB*) were not present in the genome, although *sat* and *aprA*B genes, whose enzyme products catalyze the reversible transformation of sulfate to sulfite, were detected (Table 2).

### Transporters

**Figure 5:**
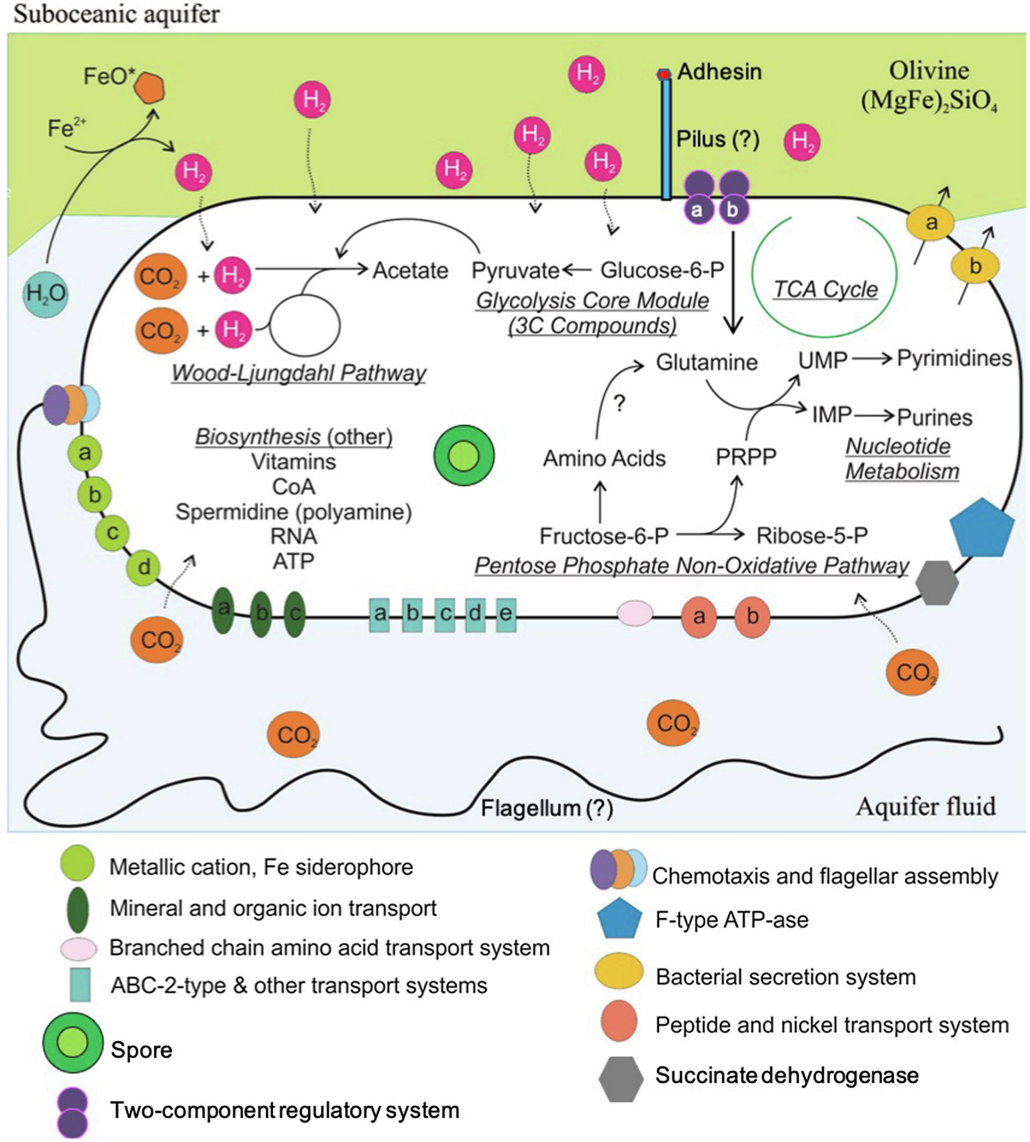
Metabolic pathways of *Ca.* A. pyornia. This organism contains the complete Wood–Ljungdahl pathway for carbon fixation, hydrogenases, and no carbohydrate or lipid importers. It is predicted that this organism is able to take advantage of hydrogen production on Fe^2+^-bearing minerals like olivine as they react with seawater. Hydrogen and bicarbonate can be funneled into the Wood–Ljungdahl pathway to produce acetyl-CoA for biosynthesis and energy. This organism is missing many amino acid synthesis pathways and likely needs to import these for biosynthesis or energy. Individual transport or regulatory systems pathways (denoted by lower case letters here) are listed in Tables 2–4 and Supplementary Tables 1–3 under the headings that match the labels in this figure.

Genes encoding transporters for proteinaceous compounds (e.g., oligopeptide, peptide/nickel, and branched chain amino acid transporters), minerals, and metals were exclusively found within the *Ca.* A. pyornia genome (Supplementary Tables 2, 3; Figure 5). When coupled with a cytosolic aminotransferase, the branched chain amino acid transport system allows for the uptake and utilization of leucine, isoleucine, and valine. *Candidatus* A. pyornia also encodes one of three enzymes that make up the branched-chain α-keto acid dehydrogenase complex, involved in the degradation of branched-chain amino acids to produce ATP. There were also complete pathways for iron, nickel, molybdate, and tungstate transporters, some of which are cofactors required for Wood–Ljungdahl pathway and respiratory enzymes.

*Candidatus* A. pyornia appears unable to import lipids or carbohydrate molecules from the environment, since genes encoding complete transporters were missing from its genome. These include the saccharide, polyol, and lipid transport system and sugar-importing phosphotransferase (PTS) systems (Supplementary Table 2).

Genes involved in export of lipooligosaccharides, heme (used in membrane-bound succinate dehydrogenase), and other components related to the cell membrane or extracellular structures were detected (Supplementary Table 2). The Sec secretion system, involved in the secretion of proteases, toxins, and pilus proteins like adhesin, was present in all acetogens compared in this study. Pathways for export of some lipooligosaccharides, which are likely membrane components, and heme, which is an integral part of the membrane-bound succinate dehydrogenase complex, were also present in the genome.

### Central Carbohydrate Metabolism

*Candidatus* A. pyornia may be capable of glycolysis *via* an alternative route to the canonical pathway. The glycolysis pathway is missing the step performed by fructose bisphosphate aldolase (Table 3), which converts α-D fructose 1,6-bisphosphate to D-glyceraldehyde 3-phosphate; however, the reverse of this step is not reported missing in the gluconeogenesis pathway, as *Ca.* A. pyornia contains the fructose 1, 6-bisphosphate aldolase, phosphatase. The gluconeogenesis pathway was deemed incomplete since the conversion of oxaloacetate to phosphoenolpyruvate *via* PEP carboxykinase is missing, but this organism encodes the oxaloacetate decarboxylase and pyruvate orthophosphate–dikinase enzymes, which provide an alternative route to gluconeogenesis (Figure 4). These enzymes enable the conversion of oxaloacetate to pyruvate and then to PEP, from which gluconeogenesis can then proceed. There are complete pathways for the glycolysis 3-C compound core module and the non-oxidative pentose phosphate pathway. The pentose phosphate pathway allows this organism to produce 5-carbon compounds for biosynthesis, and the gluconeogenesis pathway allows it to produce 6-carbon compounds. *Moorella thermoacetica*, *Ca.* D. audaxviator, and *D. hafniense* all have complete pathways for glycolysis and the pentose phosphate pathway (Table 3).

## Discussion

### Piecing Together the Genomic Puzzle

Novel genomic evidence from this study suggests that *Ca.* A. pyornia is a thermophilic acetogen from the bacterial order Clostridiales. Previously we found evidence that *Ca.* A. pyornia is a dominant member from a mineral biofilm collected from a deep subsurface oceanic crust sample (estimated to be ~15% of the community; Smith et al., 2016). This organism has the genetic potential to act as the dominant primary producer of this simple microbial community (Smith et al., 2019) and appears to be capable of chemosynthesis and energy generation using molecular hydrogen as a reductant to fix inorganic carbon in seawater. It may be supplementing its energy by utilizing amino acids or peptides commonly found in the mineral-attached biofilms, but it does not appear capable of sulfate reduction or importing carbohydrates, as has been observed for other acetogenic Clostridiales.

The full annotated MAG of *Ca.* A. pyornia is most similar to terrestrial acetogens *M. thermoacetica* and *Ca.* D. audaxviator; however, the genome of *Ca.* A. pyornia is not complete and can complicate pathway reconstruction efforts and hinder our understanding of its role in the subsurface biosphere. Further, this MAG was obtained from a flow cell loaded with crystals incubated in a subseafloor borehole rather than directly obtained from the pristine basaltic aquifer. The potential for contamination from surrounding fluids or *via* drilling processes adds to the uncertainty in this study. Nonetheless, the evolutionary relationships among acetogens in this clade provide strong evidence that this MAG represents a thermophilic organism adapted to a deep marine subsurface environment and is capable of performing acetogenesis using molecular hydrogen and CO_2_.

### Evolutionary Relationship to the Acetogen Firmicutes Clade

The phylogenetic relationships of *Ca.* A. pyornia to other acetogens suggest that this is a novel organism within a clade of related acetogens (Figure 1; Supplementary Figure 1). This group now contains both marine and terrestrial organisms; however, the marine acetogens from oceanic crust have only recently been described (Jungbluth et al., 2017; Smith et al., 2019). The terrestrial acetogen *Ca.* D. audaxviator was first described from deep continental crust (Chivian et al., 2008), *D. hafniense* from contaminated soil (Nonaka et al., 2006), and *M. thermoacetica* from horse manure (Fontaine et al., 1942). A new marine acetogen related to *Ca.* D. audaxviator, *Ca.* Desulfopertinax cowenii, was recently described from the JdFR flank aquifer fluid community (Jungbluth et al., 2017), and seven other putative acetogens were discovered from the JdFR olivine community, one of which is the *Ca.* A. pyornia MAG described here. *Candidatus* D. audaxviator and *M. thermoacetica* appear to be the closest genomic relatives to *Ca.* A. pyornia that are known acetogens; however, their genome similarity to *Ca.* A. pyornia does not exceed 80%. We are proposing *Ca.* A. pyornia represents at the very least a new genus within the Clostridiales.

### A Biofilm-Adapted Primary Producer?

The presence of this acetogen could impact carbon cycling and the health and function of the suboceanic aquifer ecosystem and the deep ocean by fixing carbon that makes its way back to the seafloor (Mason et al., 2009; McCarthy et al., 2010). *Candidatus* A. pyornia may be a prominent member of the attached community in Hole 1301A (and perhaps 1026B) as evidenced by its ubiquity in colonized rock and mineral biofilms (Orcutt et al., 2011; Smith et al., 2016) and because it encodes the full Wood–Ljungdahl carbon fixation pathway (Smith et al., 2019; Figure 2). Additionally, olivine-rich crust in this same region of the seafloor was previously revealed as a habitat for active methane- and sulfur-cycling microbes (Lever et al., 2013). This organism’s genome also encodes reductases and a ferredoxin that may be used to generate ATP *via* oxidative phosphorylation (Pierce et al., 2008). Since this organism has only been described from mineral biofilms and does not appear to be a dominant member of the aquifer fluid community, the organism this genome represents has indications that it benefits from a mineral biofilm lifestyle. A mineral biofilm can provide a wider range of substrates for metabolism either obtained from the mineral as it reacts with water or from other members of the community. Since H_2_ can be produced by mineral surfaces as they react with water in the thermal aquifer (Mayhew et al., 2013), this organism may be using H_2_ to power the Wood–Ljungdahl pathway. Charged mineral surfaces can also attract other charged molecules like certain amino acids from aquifer fluid (Lin et al., 2015), effectively concentrating these nutrients near the mineral surface. There is also some indication that this organism may participate in a syntrophic relationship in the biofilm community, especially since *Ca.* A. pyornia is missing some complete biosynthetic pathways, such as those involved in amino acid biosynthesis, and is able to import small peptides (Table 4; Supplementary Table 2).

### *acs* Gene Cluster Comparison

The co-localization of *acs* genes within a gene clusters is a common attribute of acetogens and may allow this pathway to be horizontally transferred (Pierce et al., 2008). Previously, it was found that *acs* gene clusters come in several varieties (Pierce et al., 2008), and the gene arrangement of *Ca.* A. pyornia, along with the gene cluster that most closely resembles that of *Ca.* A. pyornia (*Ca.* D. audaxviator), may form a new class of *acs* gene cluster that is unlike those that were described before. The gene clusters of *Ca.* A. pyornia and *Ca.* D. audaxviator are near-identical, with the only difference being the annotation for the 4Fe-4S ferredoxin (green arrow, Figure 3). This gene was annotated as *hdrA*, also a 4Fe-4S ferredoxin, but these genes may represent isoforms of the same heterodisulfide reductase.

The *acs* gene clusters of *M. thermoacetica* and *D. hafniense* are similar to the *Ca.* A. pyornia and *Ca.* D. audaxviator gene clusters in that they share the same *acs* genes in the same arrangement (Figure 3); however, there are some marked differences with each genome. *Moorella thermoacetica*’s genome contains another heterodisulfide reductase gene (*hdrC*) and a hypothetical protein (hp) after the *acsE* gene, and *D. hafniense* has a simplified gene cluster with no other associated genes after *acsE*. Both *M. thermoacetica* and *D. hafniense* also contain an *acsF* gene at the beginning of the *acs* cluster (Figure 3). *Moorella thermoacetica* also contains an unannotated isoform of the acsF gene within its cluster, the cobyrinic acid a,c-diamide synthase gene. Due to the similarity of *Ca.* A. pyornia’s *acs* gene cluster with that of *Ca.* D. audaxviator’s, it is likely that these organisms have a shared history relating to the Wood–Ljungdahl pathway, and these gene clusters may form a new cluster arrangement apart from the one *M. thermoacetica* or *D. hafniense* occupies.

### Incomplete TCA Cycle

*Candidatus* A. pyornia contains an incomplete TCA cycle and is missing citrate synthase and malate dehydrogenase. Although incomplete TCA cycles are common in Clostridia, such as the closely related acetogens discussed here (Nonaka et al., 2006; Chivian et al., 2008; Pierce et al., 2008), the missing enzymes vary. All of the bacteria from the olivine community to which *Ca.* A. pyornia belongs (eight microbes – two “Bacteria,” six Clostridia) were also missing citrate synthase from their genomes, and the acetogens *Ca.* D. audaxviator and *D. hafniense* are also missing this enzyme. It is not known whether *Ca.* A. pyornia can make citrate using alternative pathways. *Candidatus* A. pyornia is also missing malate dehydrogenase, as is *M. thermoacetica* (Pierce et al., 2008); however, *Ca.* A. pyornia should still be able to make oxaloacetate through the aspartate aminotransferase reaction (Figure 4). It is possible this is a sign of genome reduction in the deep subsurface, but since it appears widespread in this biofilm community, this reduction would have had to have taken place long ago in an ancestral lineage. Alternatively, the ancestral organisms in this environment never evolved to have them and this absence of TCA cycle steps was the ancestral form.

The other closely related acetogens to *Ca.* A. pyornia are also missing some TCA cycle genes; however, these varied among genomes. As noted above, among those that shared the same missing enzymes as *Ca.* A. pyornia, *Ca.* D. audaxviator and *D. hafniense* are both missing citrate synthase (Nonaka et al., 2006; Chivian et al., 2008), and *M. thermoacetica* is missing malate dehydrogenase (Pierce et al., 2008). *Candidatus* D. audaxviator is missing the additional enzymes aconitase and α-ketoglutarate dehydrogenase, *M. thermoacetica* is also missing succinyl-CoA synthetase and fumarase, and *D. hafniense* is missing isocitrate dehydrogenase and succinate dehydrogenase. *Candidatus* D. audaxviator and *D. hafniense* do contain fumarate reductase that can act similarly to succinate dehydrogenase; thus, these organisms can still complete the reversible enzymatic step that forms fumarate from succinate, depending on the conditions present (Nonaka et al., 2006; Chivian et al., 2008). However, *Ca.* A. pyornia is the only one of this group that contains all four succinate dehydrogenase genes that code for different subunits (Smith et al., 2019).

### Oxidative Phosphorylation

*Candidatus* A. pyornia may use oxidative phosphorylation during anaerobic respiration with formate as a terminal electron acceptor. *Candidatus* A. pyornia contains the genes for succinate dehydrogenase (sdhABCD), and heme export (Complex II) has all but one gene coding for NADH dehydrogenase (Complex I) and has the complete gene set for production of the F-type ATPase (Supplementary Table 1). This genome also contains abundant hydrogenases, ferredoxins, and cytochromes that may be used to shuttle protons out of the cell to create a proton-motive force and drive oxidative phosphorylation (Vignais and Billoud, 2007; Smith et al., 2019). These electron transport proteins may be coupled to the Wood–Ljungdahl pathway to provide additional energy for the cell, as has been proposed in *M. thermoacetica* (Pierce et al., 2008; Schuchmann and Müller, 2014). All genomes from the olivine community from which *Ca.* A. pyornia originated lacked at least one gene to complete NADH dehydrogenase, but most of the 11 high-quality genomes lacked more than one gene. *Candidatus* A. pyornia and two other genomes lacked only one gene, and one of these was missing the gene that codes for the same subunit as *Ca.* A. pyornia, *nuoG*. This organism is not an acetogen and is unrelated as it belongs to Archaea. Although these *nuo* genes were typically found in clusters of two or more throughout the olivine community genomes and were often associated with Wood–Ljungdahl pathway genes, there did not appear to be a conserved operon with potential un-annotated or mis-annotated *nuo* genes. There is evidence that formate dehydrogenase and some NAD(P)^+^-dependent hydrogenases contain the *nuoEFG* module of NADH dehydrogenase (Friedrich and Scheide, 2000), so perhaps *Ca.* A. pyornia is able to couple these enzymes together for respiration or use an alternative module to complete the NADH dehydrogenase complex. The NADH dehydrogenase complex has also been shown to be coupled with hydrogenases such as *ech*, and *Ca.* A. pyornia has this and a myriad of other hydrogenases that may be candidates for such coupling (Smith et al., 2019). Thus, it remains unknown whether *Ca.* A. pyornia is able to synthesize a fully functional NADH dehydrogenase, but there may be a mechanism to allow it to complete anaerobic respiration. In contrast to the other acetogens discussed here, the deep subsurface *Ca.* D. audaxviator only has the *nuoEFG* complex, but *M. thermoacetica* and *D. hafniense* contain the complete gene set (*nuoA*–*nuoN*) to synthesize this complex.

### Lack of Sulfate Reduction and Carbohydrate/Lipid Degradative Pathways

*Candidatus* A. pyornia does not appear to use sulfate reduction, carbohydrates, or lipids for energy, although key genes may have been either unannotated or mis-annotated in our analysis. This may be indicated a loss of function due to competition with others for sulfate or a possible syntrophic relationship whereby less energy can be devoted to producing substrates that can be obtained from others nearby. Although the *Ca.* A. pyornia genome is high-quality and is nearly complete, we are missing 8.5% of its genome that may contain key metabolism genes. The closely related *Ca.* D. audaxviator is a sulfate reducer that uses nitrogen fixation and carbohydrate degradation to supplement its energy (Chivian et al., 2008). *Moorella thermoacetica* also uses carbohydrates (Pierce et al., 2008), and among other metabolic strategies, *D. hafniense* is known to dechlorinate compounds for energy (Nonaka et al., 2006). Since *Ca.* A. pyornia is unable to use these substrates and energy sources for supplemental metabolism, it may be using other substrates that are not typical of the other acetogens.

### Transport and Degradation of Amino Acids and Oligopeptides

Although all acetogens considered in this study can import branched-chain amino acids, oligopeptides, and contain a peptide/nickel transporter, *Ca.* A. pyornia may rely on these compounds as a source of energy. *Candidatus* A. pyornia had more genes related to respiration and amino acid metabolism in its genome than any other organism in its community (4.86 and 9.17%, respectively), as well as a larger number of peptidases and proteases. This suggests that *Ca.* A. pyornia is either more dependent on amino acids and peptides as an additional source of energy than other members of its community or that it imports these compounds to spend less energy in producing them.

Dissolved amino acids are available in the JdFR aquifer, although their concentrations are less than typical submarine hydrothermal fluid and are instead roughly equivalent with concentrations in deep seawater (1–13nM for dissolved free amino acids and 43–89nM for dissolved hydrolyzable amino acids; Lin et al., 2015). The lower amino acid concentrations in the JdFR basement fluids relative to seawater could indicate interaction with adsorptive mineral surfaces as has been suggested (Lin et al., 2015), or it could be an indication of consumption by the microbial community. *Candidatus* A. pyornia could be one such organism contributing to this drawdown, and since it was detected here in mineral biofilms, it may be utilizing those amino acids adsorbing to the mineral surfaces as an energy or nitrogen source. For *Ca.* A. pyornia, it would be beneficial to use amino acid fermentation in this environment, especially since this organism does not appear to use or import other exogenous substrates, and using an uncommon metabolic strategy (Fonknechten et al., 2010) may mean less competition.

Although the pathways to import and begin the degradation of branched-chain amino acids are present in the *Ca.* A. pyornia genome, the degradation pathway is incomplete. It is possible that the fate of these amino acids lies in Stickland fermentation; however, we were unable to identify enzymes that could be used to ferment amino acids using this strategy. Stickland fermentation couples the oxidation and deamination of one amino acid to the reduction and deamination of another (Nisman, 1954; Fonknechten et al., 2010), producing ATP during a subsequent enzymatic reaction involving acetylphosphate. *Candidatus* A. pyornia contains the enzymes alanine dehydrogenase and glycine dehydrogenase (Supplementary Table 5) to complete the deamination reaction; however, we could not identify an amino acid reductase that these could be coupled with. Glycine dehydrogenase is coupled with glycine hydroxymethyltransferase in the interconversion of glycine and serine during the production of a Wood–Ljungdahl pathway intermediate 5,10-methylene THF (Fonknechten et al., 2010). Alanine dehydrogenase will deaminate alanine and convert it to pyruvate, while producing NADH and H^+^. The NADH and H^+^ are usually shuttled to an amino acid reductase to deaminate the other amino acid to acetylphosphate and then acetate. ATP is produced from each amino acid in the Stickland reaction when acetylphosphate is converted to acetate; therefore, it could be used as an alternative energy source for acetogens in environments like the suboceanic aquifer where carbohydrates and lipids are scarce.

### Central Carbohydrate Metabolism

*Candidatus* A. pyornia may be able to build 5- and 6-carbon sugars for biosynthesis of saccharides, nucleotides, and energy and enzymatic cofactors *via* a modified gluconeogenesis pathway originating from pyruvate. The enzyme pyruvate, orthophosphate kinase allows for the conversion of pyruvate to phosphoenolpyruvate that is one of the steps of the Crassulacean acid metabolism (CAM) carbon fixation pathway (Table 2; Supplementary Table 5; Figure 4). *Candidatus* A. pyornia is unable to complete glycolysis, which is unusual among the closely related acetogens (Table 3); however, the lack of sugar importers along with the missing genes for the glycolysis pathway suggests that *Ca.* A. pyornia is not utilizing exogenous carbohydrates and must therefore synthesize these biomolecules from pyruvate using the modified gluconeogenesis pathway.

### Other Carbon Metabolisms

*Candidatus* A. pyornia is able to synthesize nucleotides and some amino acids by using the non-oxidative pentose phosphate pathway (Table 3); however, the pathway for lipid synthesis is incomplete. *Candidatus* A. pyornia is missing the gene necessary to make acetyl-CoA carboxylase (ACC), which forms malonyl-CoA from acetyl-CoA; however, this initiation of fatty acid synthesis is also incomplete in the other closely related acetogens (Table 3). All of these acetogens are able to elongate fatty acids, so they may have an unknown pathway for malonyl-CoA synthesis.

## Conclusion

*Candidatus* A. pyornia represents a novel deep-sea thermophilic acetogen that may be utilizing amino acids instead of carbohydrates or sulfate reduction to supplement its carbon and energy metabolism. Its prevalence in this subseafloor-incubated olivine community is unusual in that its acetogenic metabolism is not predicted under thermodynamic considerations. This organism’s products may be a source of carbon for others in the community, particularly since it is capable of chemoautotrophy and produces acetate as a waste product. *Candidatus* A. pyornia’s genome indicates that this organism is uniquely suited to life on Fe-bearing minerals in the subseafloor, potentially utilizing molecular hydrogen and amino acids adsorbed to mineral surfaces. Its metabolic pathways include the complete Wood–Ljungdahl pathway for acetogenesis, and it utilizes enzymes from other partial carbon fixation pathways including the reverse TCA cycle, the CAM light reaction in photosynthesis, and the reductive pentose phosphate cycle. The unusual metabolisms of *Ca.* A. pyornia have been previously undetected in the suboceanic crustal aquifer and may bring new light to the function of this vast ecosystem.

## Data Availability Statement

The datasets presented in this study can be found in online repositories. The names of the repository/repositories and accession number(s) can be found in the article.

## Author Contributions

AS and RM analyzed the genome and metabolic pathways for A. pyornia. FC and MF proposed and guided the subseafloor sampling strategy. RM, FC, and MF provided advising and dissertation support for AS as committee members and advisors. AS wrote the manuscript. All authors provided feedback and edits to the manuscript. All authors contributed to the article and approved the submitted version.

## Funding

Metagenome sequencing was made possible by the Deep Carbon Observatory Census of Deep Life supported by the Alfred P. Sloan Foundation and was performed at the Marine Biological Laboratory (Woods Hole, MA, United States). This work was funded by NASA grant NNX08AO22G and a graduate fellowship from the NSF Center for Dark Energy Biosphere Investigations. The flow cells were funded under J0972A from the U.S. Science Support Program of Joint Oceanographic Institutions.

## Conflict of Interest

The authors declare that the research was conducted in the absence of any commercial or financial relationships that could be construed as a potential conflict of interest.

## Publisher’s Note

All claims expressed in this article are solely those of the authors and do not necessarily represent those of their affiliated organizations, or those of the publisher, the editors and the reviewers. Any product that may be evaluated in this article, or claim that may be made by its manufacturer, is not guaranteed or endorsed by the publisher.

## Supplementary Material

Supplementary Figure 1 *ffh* gene phylogeny of *Ca*. Acetocimmeria pyornia and closest relatives. The evolutionary history of *Ca*. Acetocimmeria pyornia was inferred using the Maximum Likelihood method based on the Tamura-Nei model in MEGA5 (Tamura et al., 2011), with the optimal tree for the *ffh* gene shown. The percentage of 500 replicate trees in which the associated taxa clustered together (bootstrap test) is shown next to the branches if greater than 50% (Felsenstein, 1985). Branch lengths are measured in the number of substitutions per site. There were 264 positions in the final dataset. Organisms in green are known acetogens.
