# Supplementary material for: Ancient Metabolisms of a Thermophilic Subseafloor Bacterium
Source: Front Microbiol. 2021 Dec 1;12:764631. doi: 10.3389/fmicb.2021.764631 (PMC8671834; doi:10.3389/fmicb.2021.764631)
Supplement: Supplementary file 1 [file Table_1.DOCX]

**Supplementary Table 1. Complete KEGG pathways for secondary metabolism, energy metabolism and genetic information processing.** Closely-related known acetogens: Mta = *Moorella thermoacetica*, Dau = *Ca*. Desulforudis audaxviator, and Dsy = *Desulfitobacterium hafniense*. + = complete pathway, (+) = known complete pathways not identified through the KEGG module. (-) = near-complete, with at least one gene.

| **KEGG Gene** | **Discrete pathway** | **Organism** | | | |
| --- | --- | --- | --- | --- | --- |
| **Secondary metabolism – structural complex** | | Mta | Dau | Dsy | *Ca.* Apy |
| **Energy metabolism** | |  |  |  |  |
| **ATP synthesis** | |  |  |  |  |
| M00144 | NADH:quinone oxidoreductase, prokaryotes | **+** |  | **+** | (-) |
| M00149 | Succinate dehydrogenase, prokaryotes |  |  |  | **+** |
| M00153 | Cytochrome d ubiquinol oxidase | **+** |  | **+** |  |
| M00157 | F-type ATPase, prokaryotes and chloroplasts | **+** | **+** | **+** | **+** |
| **Genetic information processing** | |  |  |  |  |
| **DNA polymerase** | |  |  |  |  |
| M00260 | DNA polymerase III complex, bacteria | **+** | **+** | **+** | (-) |
| **RNA polymerase** | |  |  |  |  |
| M00183 | RNA polymerase, bacteria | **+** | **+** | **+** | **+** |
| **Ribosome** | |  |  |  |  |
| M00178 | Ribosome, bacteria | **+** | **+** | **+** | (-) |
