# Supplementary material for: Ancient Metabolisms of a Thermophilic Subseafloor Bacterium
Source: Front Microbiol. 2021 Dec 1;12:764631. doi: 10.3389/fmicb.2021.764631 (PMC8671834; doi:10.3389/fmicb.2021.764631)
Supplement: Supplementary file 2 [file Table_2.DOCX]

**Supplementary Table 2. Complete KEGG pathways for secondary metabolism, transport systems.** Closely-related known acetogens: Mta = *Moorella thermoacetica*, Dau = *Ca*. Desulforudis audaxviator, and Dsy = *Desulfitobacterium hafniense*. + = complete pathway, (+) = known complete pathways not identified through the KEGG module. (-) = near-complete, with at least one gene.

| **KEGG Gene** | **Discrete pathway** | **Organism** | | | |
| --- | --- | --- | --- | --- | --- |
| **Secondary metabolism – environmental information processing** | | Mta | Dau | Dsy | *Ca.* Apy |
| **Mineral and organic ion transport system** | |  |  |  |  |
| M00185 | Sulfate transport system |  |  | **+** |  |
| M00189 | Molybdate transport system | **+** | **+** | **+** | **+** |
| M00186 | Tungstate transport system | **+** | **+** |  | **+** |
| M00188 | NitT/TauT family transport system | **+** |  | **+** | **+** |
| M00436 | Sulfonate transport system |  |  | **+** |  |
| M00299 | Spermidine/putrescine transport system |  |  | **+** |  |
| M00208 | Glycine betaine/proline transport system |  |  | **+** |  |
| M00209 | Osmoprotectant transport system | **+** |  | **+** |  |
| **Saccharide, polyol, and lipid transport system** | |  |  |  |  |
| M00201 | alpha-Glucoside transport system |  |  | **+** |  |
| M00207 | Putative multiple sugar transport system |  |  | **+** |  |
| M00212 | Ribose transport system | **+** |  |  |  |
| M00221 | Putative simple sugar transport system | **+** |  |  |  |
| M00211 | Putative ABC transport system |  | **+** |  |  |
| **Phosphate and amino acid transport system** | |  |  |  |  |
| M00222 | Phosphate transport system | **+** | **+** | **+** | (-) |
| M00589 | Putative lysine transport system |  |  | **+** |  |
| M00237 | Branched-chain amino acid transport system | **+** | **+** | **+** | **+** |
| M00238 | D-Methionine transport system |  |  | **+** |  |
| M00228 | Putative glutamine transport system |  |  | **+** |  |
| M00236 | Putative polar amino acid transport system | **+** | **+** | **+** |  |
| **Peptide and nickel transport system** | |  |  |  |  |
| M00439 | Oligopeptide transport system |  |  | **+** | **+** |
| M00239 | Peptides/nickel transport system |  | **+** | **+** | **+** |
| **Metallic cation, iron-siderophore and vitamin B12 transport system** | |  |  |  |  |
| M00240 | Iron complex transport system | **+** | **+** | **+** | **+** |
| M00242 | Zinc transport system | **+** |  | **+** |  |
| M00319 | Manganese/zinc/iron transport system |  |  | **+** |  |
| M00245 | Cobalt/nickel transport system | **+** | **+** | **+** | **+** |
| M00243 | Manganese/iron transport system |  |  | **+** |  |
| M00246 | Nickel transport system | **+** | **+** | **+** | **+** |
| M00247 | Putative ABC transport system |  |  | **+** |  |
| M00582 | Energy-coupling factor transport system | **+** |  | **+** | **+** |
| **ABC-2 type and other transport systems** | |  |  |  |  |
| M00298 | Multidrug/hemolysin transport system |  |  | **+** |  |
| M00813 | Lantibiotic transport system |  |  | **+** |  |
| M00762 | Copper-processing system |  |  | **+** |  |
| M00224 | Fluoroquinolone transport system |  |  | + |  |
| M00252 | Lipooligosaccharide transport system | **+** |  |  | **+** |
| M00256 | Cell division transport system | **+** | **+** | **+** | **+** |
| M00259 | Heme transport system |  |  |  | **+** |
| M00258 | Putative ABC transport system | **+** | **+** | **+** | **+** |
| M00254 | ABC-2 type transport system | **+** | **+** | **+** | **+** |

| **Drug efflux transporter/pump** | |  |  |  |  |
| --- | --- | --- | --- | --- | --- |
| M00707 | Multidrug resistance, SmdAB/MdlAB transporter |  |  | **+** |  |
| M00712 | Multidrug resistance, efflux pump YkkCD |  |  | **+** |  |
| **Phosphotransferase system (PTS)** | |  |  |  |  |
| M00273 | PTS system, fructose-specific II component | **+** |  |  |  |
| **Bacterial secretion system** | |  |  |  |  |
| M00335 | Sec (secretion) system | **+** | **+** | **+** | **+** |
| M00336 | Twin-arginine translocation (Tat) system | **+** | **+** | **+** | **+** |
