# Supplementary material for: Ancient Metabolisms of a Thermophilic Subseafloor Bacterium
Source: Front Microbiol. 2021 Dec 1;12:764631. doi: 10.3389/fmicb.2021.764631 (PMC8671834; doi:10.3389/fmicb.2021.764631)
Supplement: Supplementary file 3 [file Table_3.DOCX]

**Supplementary Table 3. Complete KEGG pathways for other energy metabolisms and regulatory systems.** Closely-related known acetogens: Mta = *Moorella thermoacetica*, Dau = *Ca*. Desulforudis audaxviator, and Dsy = *Desulfitobacterium hafniense*. + = complete pathway, (+) = known complete pathways not identified through the KEGG module. (-) = near-complete, with at least one gene.

| **KEGG Gene** | **Discrete pathway** | **Organism** | | | |
| --- | --- | --- | --- | --- | --- |
| **Energy metabolism** | | Mta | Dau | Dsy | *Ca.* Apy |
| **Functional set** | |  |  |  |  |
| **Aminoacyl tRNA** | |  |  |  |  |
| M00360 | Aminoacyl-tRNA biosynthesis, prokaryotes | **+** | **+** | **+** | **+** |
| **Nucleotide sugar** | |  |  |  |  |
| M00362 | Nucleotide sugar biosynthesis, prokaryotes |  |  |  | **+** |
| **Environmental information processing** | |  |  |  |  |
| **Two-component regulatory system** | |  |  |  |  |
| M00454 | KdpD-KdpE (potassium transport) two-component regulatory system |  |  | **+** |  |
| M00434 | PhoR-PhoB (phosphate starvation response) two-component regulatory system |  | **+** |  |  |
| M00458 | ResE-ResD (aerobic and anaerobic respiration) two-component regulatory system |  | **+** | **+** |  |
| M00459 | VicK-VicR (cell wall metabolism) two-component regulatory system |  |  |  | **+** |
| M00478 | DegS-DegU (multicellular behavior control) two-component regulatory system | **+** |  |  | (-) |
| M00483 | NreB-NreC (dissimilatory nitrate/nitrite reduction) two-component regulatory system |  |  | **+** |  |
| M00489 | DctS-DctR (C4-dicarboxylate transport) two-component regulatory system |  |  | **+** |  |
| M00490 | MalK-MalR (malate transport) two-component regulatory system |  |  | **+** |  |
| M00492 | LytS-LytR two-component regulatory system | **+** |  |  |  |
| M00518 | GlnK-GlnL (glutamine utilization) two-component regulatory system |  |  |  | **+** |
| M00519 | YesM-YesN two-component regulatory system |  |  | **+** |  |
| M00657 | VanS-VanR (VanE type vancomycin resistance) two-component regulatory system |  |  | **+** |  |
| **Drug efflux transporter/pump** | |  |  |  |  |
| M00765 | Multidrug resistance, efflux pump Bmr |  |  | **+** |  |
