# Supplementary figures and images for: Ancient Metabolisms of a Thermophilic Subseafloor Bacterium
Source: Front Microbiol. 2021 Dec 1;12:764631. doi: 10.3389/fmicb.2021.764631 (PMC8671834; doi:10.3389/fmicb.2021.764631)

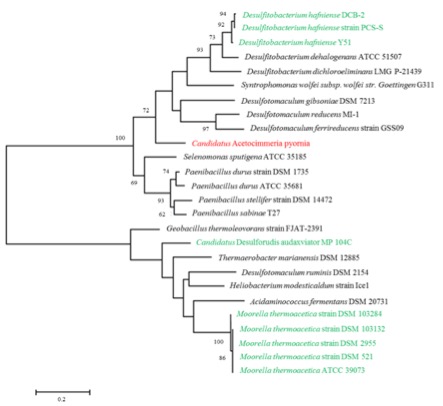

Supplement: Supplementary Figure 1 — ffh gene phylogeny of Ca. Acetocimmeria pyornia and closest relatives. The evolutionary history of Ca. Acetocimmeria pyornia was inferred using the Maximum Likelihood method based on the Tamura-Nei model in MEGA5 (Tamura et al., 2011), with the optimal tree for the ffh gene shown. The percentage of 500 replicate trees in which the associated taxa clustered together (bootstrap test) is shown next to the branches if greater than 50% (Felsenstein, 1985). Branch lengths are measured in the number of substitutions per site. There were 264 positions in the final dataset. Organisms in green are known acetogens. [file Image_1.JPEG]
